# Hyperpolarized [1‐^13^C] pyruvate as a possible diagnostic tool in liver disease

**DOI:** 10.14814/phy2.13943

**Published:** 2018-12-11

**Authors:** Uffe Kjærgaard, Christoffer Laustsen, Thomas Nørlinger, Rasmus S. Tougaard, Emmeli Mikkelsen, Haiyun Qi, Lotte B. Bertelsen, Niels Jessen, Hans Stødkilde‐Jørgensen

**Affiliations:** ^1^ MR Research Centre Aarhus University Hospital Aarhus Denmark; ^2^ Department of Cardiology Aarhus University Hospital Aarhus Denmark; ^3^ Department of Biomedicine Aarhus University Aarhus Denmark; ^4^ Department of Clinical Pharmacology Aarhus University Hospital Aarhus Denmark

**Keywords:** Glucose homeostasis, hyperpolarization, lactate dehydrogenase, liver, porcine, spectroscopy

## Abstract

Introduction of hyperpolarized magnetic resonance in preclinical studies and lately translation to patients provides new detailed in vivo information of metabolic flux in organs. Hyperpolarized magnetic resonance based on ^13^C enriched pyruvate is performed without ionizing radiation and allows quantification of the pyruvate conversion products: alanine, lactate and bicarbonate in real time. Thus, this methodology has a promising potential for in vivo monitoring of energetic alterations in hepatic diseases. Using ^13^C pyruvate, we investigated the metabolism in the porcine liver before and after intravenous injection of glucose. The overall mean lactate to pyruvate ratio increased significantly after the injection of glucose whereas the bicarbonate to pyruvate ratio was unaffected, representative of the levels of pyruvate entering the tricarboxylic acid cycle. Similarly, alanine to pyruvate ratio did not change. The increased lactate to pyruvate ratio over time showed an exponential correlation with insulin, glucagon and free fatty acids. Together, these data, obtained by hyperpolarized ^13^C magnetic resonance spectroscopy and by blood sampling, indicate a hepatic metabolic shift in glucose utilization following a glucose challenge. Our findings demonstrate the capacity of hyperpolarized ^13^C magnetic resonance spectroscopy for quantifying hepatic substrate metabolism in accordance with well‐known physiological processes. When combined with concentration of blood insulin, glucagon and free fatty acids in the blood, the results indicate the potential of hyperpolarized magnetic resonance spectroscopy as a future clinical method for quantification of hepatic substrate metabolism.

## Introduction

The liver plays a central role in regulation of whole body glucose homeostasis (Wasserman 2009). During hypoglycemia, the liver releases glucose into the circulation produced by gluconeogenesis and glycogenolysis while during hyperglycemia, the liver stores glucose as glycogen (DeFronzo et al. 1981; Ferrannini et al. 1985). These processes are tightly regulated by hormones and circulating substrate levels (Roden and Bernroider 2003). Insulin stimulates glycolysis and suppresses gluconeogenesis but promotes glucose storage in the liver as glycogen under hyperglycemia. In contrast, glucagon suppresses glycolysis and stimulates gluconeogenesis. In various diseases the liver glucose homeostasis is of pivotal importance, especially in type 2 diabetes mellitus (T2DM) and nonalcoholic fatty liver disease (NAFLD) (Roden and Bernroider 2003).

Combined with the dynamic nuclear polarization (DNP) technology, hyperpolarized magnetic resonance spectroscopy (MRS) provides organ‐specific metabolic information in a minimally invasive manner. This highly sensitive technology (more than 10,000 times above conventional MRS [Ardenkjaer‐Larsen et al. 2003]) has recently been demonstrated to allow for in vivo quantification of hepatic glycolytic pathways in rats (Kohler et al. 2007; Lee et al. 2013). It is possible to quantify the breakdown products of pyruvate with a time resolution of approximately 1 sec. This is performed by injecting a hyperpolarized bio‐active molecule, for example, ^13^C enriched pyruvate, which has had one of the ^12^C atoms substituted with the stable isotope ^13^C, while simultaneously acquiring MRS data in a predefined tissue volume in the liver (Day et al. 2007). The use of hyperpolarized bio‐probes like pyruvate, or other polarizable agents, provides an opportunity to combine the flexibility and safety of MR with metabolic, spectroscopic data recorded with an exceptionally high signal‐to‐noise ratio.

**Figure 1:**
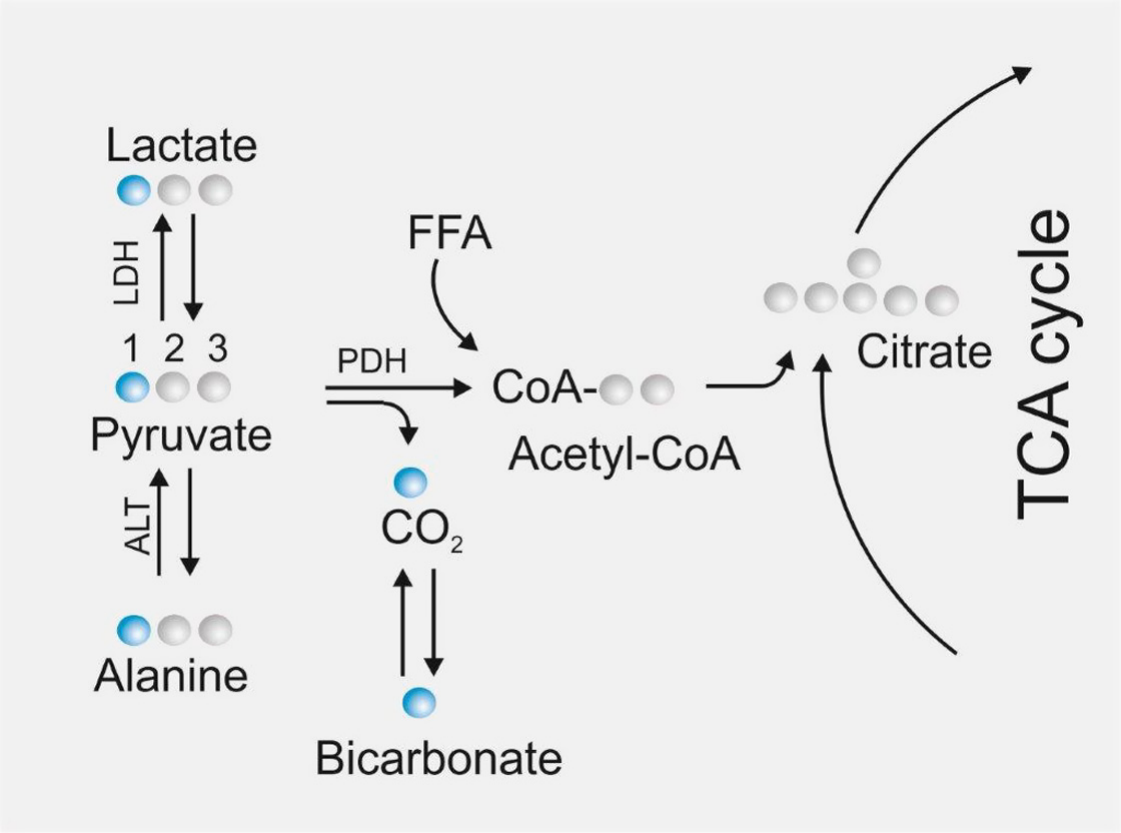
Metabolism of [1‐^13^C] pyruvate. Key metabolic reactions and conversions relevant for the tracing of [1‐^13^C]‐labeled pyruvate. Circles represent carbon atoms in the molecules where blue circles allow tracing of the first carbon atom in pyruvate where ^12^C has been exchanged with a ^13^C atom. Abbreviations: Pyruvate dehydrogenase (PDH), alanine aminotransferase (ALT), lactate dehydrogenase (LDH), free fatty acids (FFA), and tricarboxylic acid (TCA). FFA are converted to acetyl‐CoA via beta‐oxidation, which enters the TCA cycle.

At present, the most commonly used agent for DNP is ^13^C pyruvate (Zierhut et al. 2010; Laustsen et al. 2013; Hansen et al. 2017; Tougaard et al. 2018). This substrate can be traced in its metabolites alanine, bicarbonate and lactate (Fig. 1). The enzymes responsible for these conversions are: alanine aminotransferase (ALT), pyruvate dehydrogenase (PDH) and lactate dehydrogenase (LDH). LDH catalyzes the reversible process of pyruvate to lactate where nicotinamide adenine dinucleotide hydrate (NADH_2_) is oxidized to nicotinamide adenine dinucleotide (NAD^+^). The catalytic conversion by LDH is primary substrate dependent , but epinephrine induces the conversion of lactate to pyruvate, whereas insulin suppresses this conversion (Adeva et al. 2013; Fiume et al. 2014).

ALT catalyzes the reversible reaction of pyruvate and glutamate to *α*‐ketoglutarate and alanine. Both LDH and ALT are cytosolic enzymes, while the PDH‐complex is present in the mitochondrial matrix. PDH converts pyruvate and Coenzyme A (CoA) irreversibly to Acetyl‐CoA and CO_2_ while reducing NAD^+^ to NADH_2_ (Adeva et al. 2013).

This means that dynamic quantification of lactate metabolized from pyruvate is an indirect estimate of the factors involved in the reaction, for example, lactate, pyruvate, NAD+, NADH2 as well as LDH activityin the specific organ in question. Furthermore, owing to the bicarbonate released when pyruvate is converted to Acetyl‐CoA, which can also be quantified, information about aerobic pyruvate usage can be estimated as well.

During the last decade, a large number of DNP studies based on injectable ^13^C‐labeled substances have been conducted on rodents (Merritt et al. 2011; Moreno et al. 2014; Josan et al. 2015; Jin et al. 2016; Kim et al. 2016; Moon et al. 2017; Park et al. 2017) and, more recently, also on patients with prostatic cancer and healthy subjects to study the heart (Nelson et al. 2013; Cunningham et al. 2016). In this study, a porcine model was chosen, as the large animal model's robustness to fasting conditions and the tolerance to consecutive injections of [1‐^13^C] pyruvate, means that the large animals present a viable translational approach in to human studies. Furthermore, the large animal model allows clinical routine examinations such as blood sampling for biochemistry assays to be collected under clinical‐like conditions. To date, few studies have been performed on larger animals, and to the best of our knowledge, this study is the first to demonstrate a large animal hepatic examination using hyperpolarized MRS. A direct obstacle in the adaptation of this method in the daily clinical practices, is the qualitative nature of the hyperpolarized MR data, yielding solely apparent rate constants. In this study, we analyzed the relationship between changes in typical blood parameters and changes in hyperpolarized MRS following a glucose challenge.

## Materials and Methods

### Animal model

Twelve anaesthetized healthy Danish female landrace pigs were included in the study; one animal, however, showed significantly lower oxygen saturation than the other pigs (data not shown) and was excluded, thus leaving eleven pigs (30.7 kg ± SD 1.3) with normophysiological appearance. The pigs were food deprived for at least four hours before the first DNP measurement. All pigs were sedated with i.m. injection of a mixture of stressnil (0.1 mL/kg bodyweight) and midazolam (2.0 mg/kg bodyweight). IV access was obtained by insertion of a size 20 G venflon in an ear vein. Each pig received 120 mg propofol (B. Braun Medical A/S, Denmark) i.v. Subsequently, it was endotracheal intubated and mechanically ventilated with a tidal volume of 300–425 mL and 12–20 breaths per min aiming at an expiratory CO_2_ of 5.5%. Anesthesia was maintained by 3% sevoflurane mixed in atmospheric air with 40% oxygen. Fentanyl (Hameln Pharmaceuticals, Hameln, Germany) was administered i.v. in the dose of 166 *μ*g/h/kg for pain relief. A urinary bladder catheter was applied. To monitor blood pressure and to obtain blood samples, a 5fr. catheter was inserted into the left femoral artery with ultrasound guidance. Additionally, two 5fr. catheters were inserted into the femoral veins, one on each side, one catheter for administration of hyperpolarized [1‐^13^C] pyruvate samples and one catheter for venous blood sampling. To avoid clotting of the intravascular catheters, heparin (100 IU/kg bodyweight) was administered i.v. At the end of the experiment, the pigs were euthanized with 100 mg/kg pentobarbital administered i.v. The study was approved by the Danish Animal Experiments Inspectorate.

### MR imaging and spectroscopy

**Figure 2:**
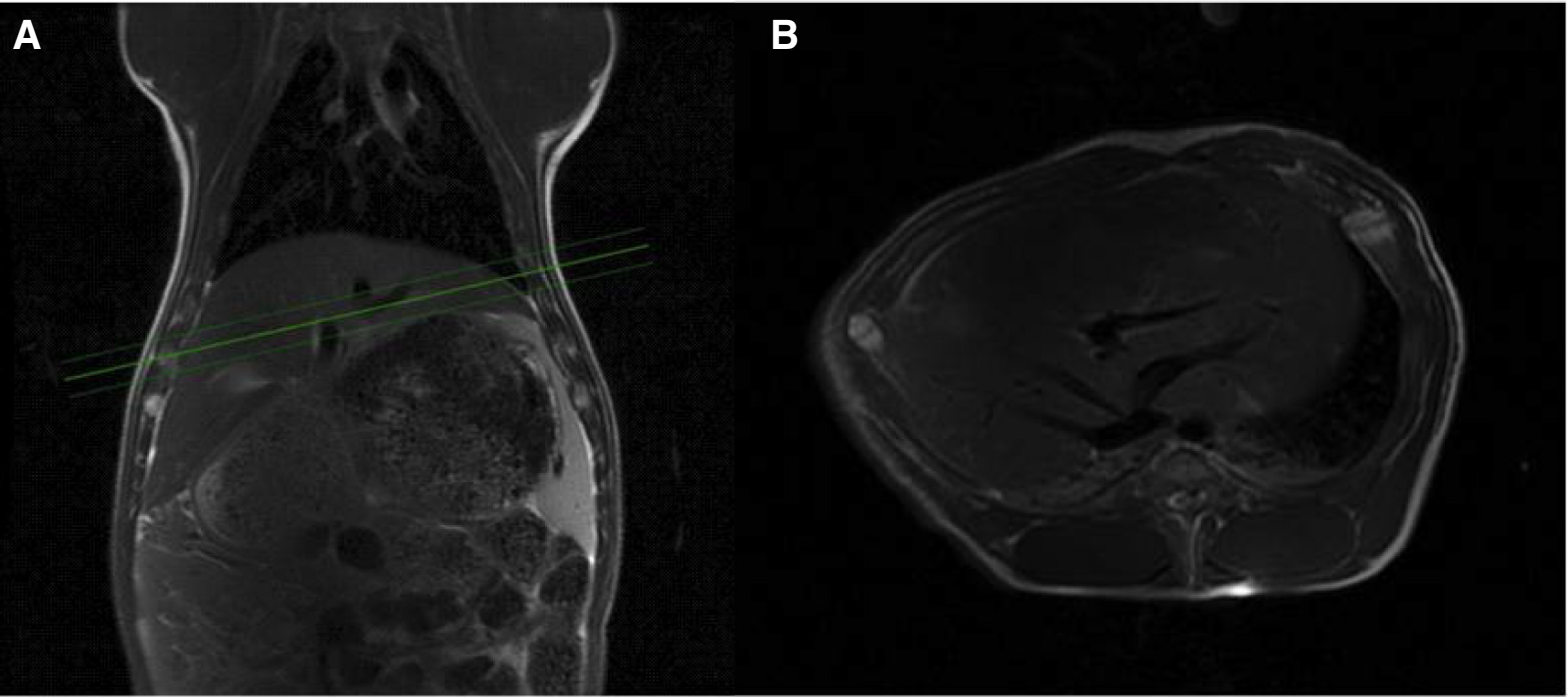
Magnetic resonance (MR) spectra and proton images. MR‐Proton images of one of the pigs, showing a coronal image (A) and oblique image through the liver (B). The three green lines show the 1 cm section for the ^13^C MRS time series.

A 3T GE Hdx MR scanner (GE Healthcare, Milwaukee, WI) was used for both MRI and hyperpolarizied MRS. An integrated body coil was used for proton imaging. Respiratory frequencies excitations were gated by the animal's respiratory cycles. Localization was performed with a standard three plane single shot fast spin echo localizer sequence, TR = 2 sec and TE = 98 ms. Subsequently, anatomical fast spin echo images were acquired with axial coronal and oblique 18 slices of 8 mm slice thickness, TR = 5 s and TE = 92 ms (Fig. 2). These images were used for locating the ^13^C slice selection across the liver in the subsequent DNP measurement. For ^13^C spectroscopy, the scanner was equipped with a bore‐insertable ^13^C volume excitation resonator integrated in the patient table (GE Healthcare). This was combined with a 16‐channel flexible receiver heart array coil (Rapid Biomedical, Rimpar, Germany) which consisted of two paddles, each with eight channels for placement underneath and on top of the animal to ensure sufficiently uniform coil sensitivity in the liver slice volume.

Prior to the ^13^C scan, a references spectrum was acquired using a Bloch sigert shift sequence to map the radiofrequency, combined with placement of a 2M ^13^C bicarbonate phantom placed in the liver region of the excitation coil. Expiratory triggered hyperpolarized spectroscopy was initiated at the start of injection and was based on a 1 cm thickness oblique single slice covering the liver; repetition time approximately 1 sec; total recording period 120 sec; flip angle 10°. The plane was manually adjusted to cover as much liver tissue as possible and minimizing other tissue, especially muscles.

The 16 channels were individually processed by the Hankel singular value decomposition method in MATLAB, and the data from each coil element were combined (Van Huffel 1993; Lauritzen et al. 2013). The peak area from bicarbonate, alanine and lactate levels were expressed as a ratio to the pyruvate signal, whereas pyruvate levels were expressed as a ratio to the sum of all ^13^C signals.

Peak quantification was performed by conventional integration for each of the peaks of interest. The metabolic derivatives of pyruvate was normalized to pyruvate to follow normal conventions in hyperpolarized ^13^C. To assess the pyruvate accumulation pyruvate was normalized to the sum of all ^13^C signal.

### Hyperpolarized sample preparation

For sample preparation, 600 mg of [1‐^13^C] pyruvic acid was mixed with 15 mmol/L of a paramagnetic agent (EPA) as previously described in Ardenkjaer‐Larsen et al. (2011). In short, dissolution was performed with 29 mL of deionized water into a receiver syringe. The receiver syringe contained 13 mL of 360 mmol/L NaOH and 180 mmol/L tris (hydroxymethyl)aminomethane (TRIS) for neutralization of the pyruvic acid. Four identical samples of the mixture of 600 mg of [1‐^13^C] pyruvic acid/ 15 mmol/L EPA were polarized in a SpinLab (GE Healthcare) for more than two hours to ensure approximately 40% polarization.

Twenty‐eight milliliter of hyperpolarized pyruvate solution (250 mmol, pH 7.4, isotonic) were injected into the pig via the left femoral vein catheter (Laustsen et al. 2015) at time 0, 20, 40 and 60 min. Five minutes after the first injection of hyperpolarized pyruvate, a bolus of 1 g/kg glucose (glucose, 500 g/L; SAD, Copenhagen, Denmark) was injected into the right femoral vein catheter over approximately 20 sec.

### Blood samples

Blood samples were collected prior to, during and after the experiment. Arterial blood samples was collected from the artery catheter and analyzed using an ABL 700 Series Blood Gas Analyzer (Radiometer Copenhagen, Denmark). Blood samples were obtained from the right femoral vein and collected into 3.5 mL tubes (BD Vacutainer^®^ SST™ II Advance^®^) to determine the levels of insulin and free fatty acids (FFA). The tubes were stored at room temperature for an hour, insulin and FFA samples were centrifuged at 2000 and 1200 ***g***, respectively, over ten minutes at room temperature. The serum was then pipetted and frozen at −20°C. Insulin was analyzed using time‐resolved fluoroimmunoassay (TR‐IFMA; AutoDELFIA, PerkinElmer, Turku, Finland), and FFA were analyzed by a commercial kit (Wako Chemicals, Neuss, Germany). Serum glucagon concentrations were determined using a radioimmunoassay (EMD Millipore's Glucagon Radioimmunoassay (RIA) Kit, Germany) following the instructions supplied by the manufacturer.

### Statistics

Normality was assessed with quantile plots. A *P* < 0.05 (*) was considered statistically significant. To take into account the repeated nature of the measurements, a repeated measurement analysis of variance (ANOVA) was used to compare the overall and individual metabolic and functional data. A Dunnett's Post hoc correction for multiple comparisons was used to examine the individual timepoints, 0 and 4 min. The exponential fitting was performed by a linear regression on the log‐transformed data using a least‐square fit. Statistical analysis was performed in GraphPad Prism (GraphPad Software, Inc., La Jolla, CA). Results are presented as mean ± SEM. No power calculation was performed as this to the best of our knowledge is the first large animal liver examination with hyperpolarized MRS.

## Results

### Liver‐specific metabolic alterations

**Figure 3:**
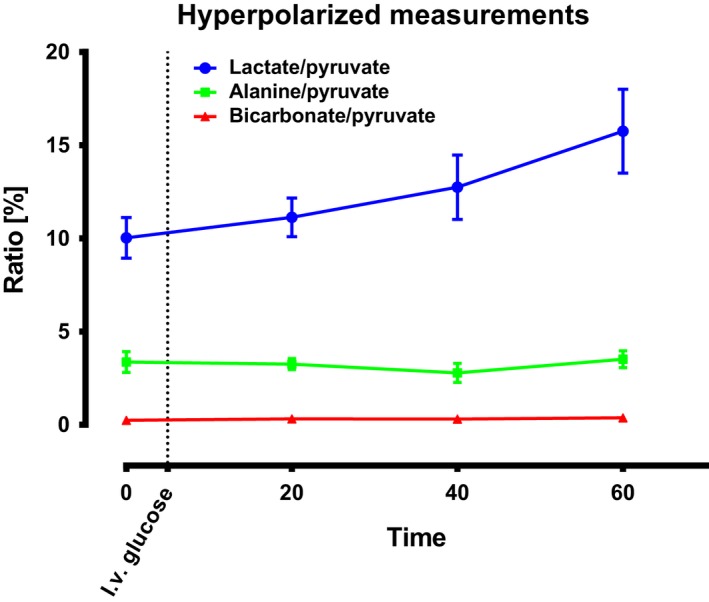
Hyperpolarized measurements. Hepatic hyperpolarized metabolic conversion increased statistically significantly in the lactate to pyruvate ratio (*P* < 0.01) following glucose challenge whereas the alanine to pyruvate ratio (*P* = 0.54) and bicarbonate to pyruvate ratio (*P* = 0.62) showed no detectable alterations. Repeated measurement analysis of variance (ANOVA) was used for testing. Sample size *N* = 11, mean ± SEM. The ratios (lactate to pyruvate ratio, bicarbonate to pyruvate ratio and the alanine to pyruvate ratio) are the sum of all the respective spectra's divided by the sum of all the pyruvate spectra's. The ratios therefore show the total conversion of pyruvate into over a 120 sec time period.

The hyperpolarized ^13^C pyruvate measurements of pyruvate to lactate ratio increased over time by 57% (Fig. 3, *P* < 0.01). The pyruvate to alanine ratio did not change over time (*P* = 0.54). Similarly, pyruvate to bicarbonate ratio (*P* = 0.62) was unaltered.

### Acute pyruvate injection effects

**Figure 4:**
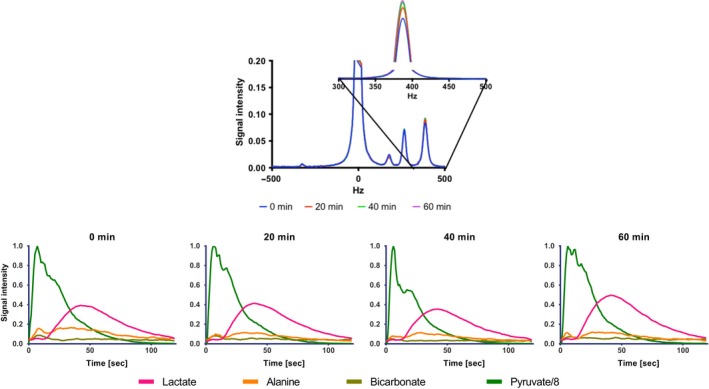
Spectra of ^13^C‐MRS. Top figure shows the frequency (Hz) each molecule has from left to right is pyruvate, bicarbonate, alanine and lactate. The enhancement of the graph shows the lactate peaks at 390 Hz. The bottom row shows four typical spectra of 120 sec of acquisition at 0 min, 20 min, 40 min and 60 min (from left to right) as a function of time. After 120 sec the signal of hyperpolarized molecules is disappeared.

**Figure 5:**
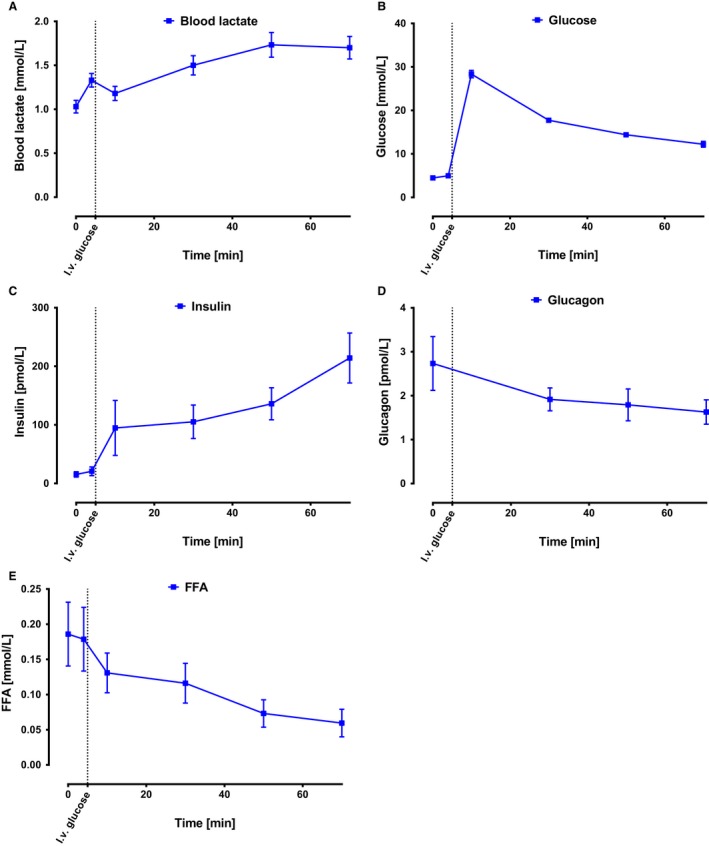
Hormones and metabolites concentrations in blood. Time course of blood metabolites and hormones (Mean ± SEM) for (A) blood lactate; *n* = 11, (B) glucose; *n* = 11 (C) insulin; *n* = 9(D) glucagon; *n* = 7, and (E) FFA;* n* = 10.

A series of four typical ^13^C‐MRS spectra and time course over the 120 sec acquisition is shown in Figure 4. FFA, glucose and insulin blood levels did not significantly change compared to the levels at time = 0 min and time = 4 min (data not shown). The blood levels of lactate increased in the same time span (*P* < 0.01). Subsequently, blood lactate decreased (at 10 min) and then increased continuously (Fig. 5E). No change in the apparent [1‐^13^C]pyruvate flow or delivery (pyruvate time to peak of 8 ± 4 sec) was seen between injections in the individual animals.

### Systemic glucose challenge effects

Following a glucose injection, blood glucose levels (Fig. 5A) increased from 4.5 ± 0.3 mmol/L at 0 min to 28.3 ± 0.84 mmol/L at 10 min (*P* < 0.01) and subsequently decreased to 12.2 ± 0.66 mmol/L at 70 min. Insulin levels (Fig. 5C) increased from 15.3 ± 4.6 pmol/L at 0 min to 214.2 ± 42.7 pmol/L at 70 min (*P* = 0.03). Glucagon (Fig. 5D) levels did not change significantly over time whereas circulating FFA levels (Fig. 5B) decreased from 0.19 ± 0.05 mmol/L to 0.060 ± 0.020 mmol/L following the glucose challenge (*P* = 0.004).

### Metabolic and hormone interactions

**Figure 6:**
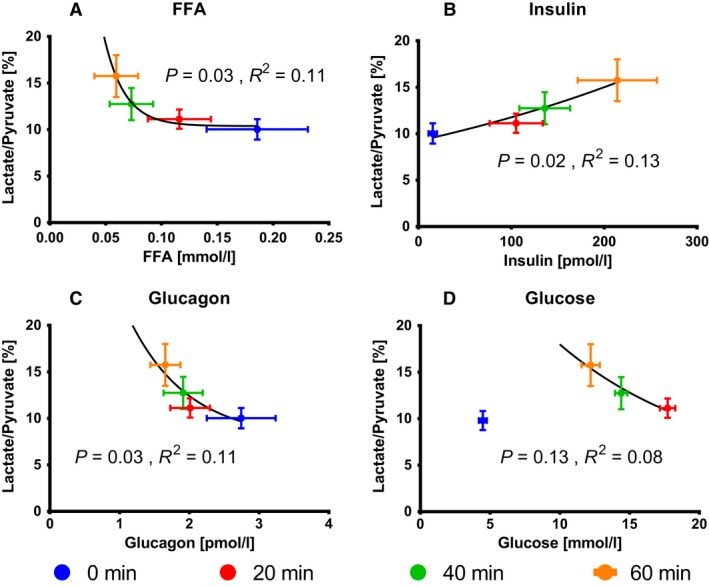
Blood concentration of hormones and metabolites combined with lactate to pyruvate ratio. The lactate to pyruvate ratio and the corresponding FFA (A), glucagon (B), insulin (C) and glucose (D) at 0, 30, 50 and 70 was fitted with a single exponential regression, showing a clear relationship for the lactate to pyruvate ratio and FFA, glucagon and insulin respectively. A similar dependency was observed by omitting the initial mean for glucose. The color code represents the time of injection of hyper polarized ^13^C pyruvate injection: blue (0 min), red (20 min), green (40 min), and orange (60 min). Sample size for glucose (*n* = 11), insulin (*n* = 9), glucagon (*n* = 7) FFA (*n* = 10).

Investigating the dependencies between lactate production and the systemic metabolic and hormone response demonstrated an inverse relationship on FFA, glucagon and insulin (Fig. 6). There was an inverse dependency with FFA and glucagon, whereas a positive dependency was observed with insulin. The alanine to pyruvate ratio and pyruvate to bicarbonate ratio did not demonstrate the same dependency neither with FFA, glucagon, insulin nor glucose (data not shown).

## Discussion

This study demonstrates that MRS based on DNP can be used to study hepatic substrate metabolism and is to our knowledge the first study to do so in a large animal model resembling human physiology. This was evident by the acutely altered lactate to pyruvate ratio concomitant with maintained bicarbonate to pyruvate ratio and alanine to pyruvate ratio following a glucose challenge test in a food deprived large animal model.

The main finding of this study was the inverse relationship of lactate to pyruvate ratio with insulin, glucagon and FFA blood levels. These changes in biomarkers are directly linking to altered glucose homeostasis (Roden and Bernroider 2003; Madiraju et al. 2014; Lewis et al. 2016; Qi et al. 2018a). Furthermore, a similar tendency was observed for blood glucose and interestingly to a lesser degree the plasma lactate levels.

The importance of these findings is highlighted by the potential for the establishment of a reference interval for the healthy liver using the association between hepatic metabolic dysfunction. Interestingly, no significant correlation was found at baseline for any combination of lactate to pyruvate ratio, alanine to pyruvate ratio and bicarbonate to pyruvate ratio with blood concentration of glucose, FFA or insulin, respectively (data not shown). A similar inverse relationship was found in the blood glucose when neglecting the baseline point of glucose (Fig. 5D), thus supporting the dependency of hormone and energy substrates levels with the liver lactate production. This supports the use of challenge test or alternatively clamp models for accurate quantitative determination of the hepatic metabolic signature using hyperpolarized MR. Usage of a hyperinsulinemic euglycemic glucose clamp is the golden‐standard method to determine endogen glucose production and quantifies insulin sensitivity under steady‐state conditions (Muniyappa et al. 2008; Wasserman 2009) taking numerous limitations into consideration, by making a more controlled metabolic environment, however require intensive procedures to establish the steady‐state conditions. Our data were obtained in a dynamic manor and not under steady‐state conditions, therefore possible delayed responses of hormones and metabolites are not taking into account. On the contrary examinations of insulin sensitivity like the intravenous glucose tolerance test (IVGTT) or the oral glucose tolerance test (OGTT) is widely used in the clinic (Muniyappa et al. 2008), and can easily, with their accessibility and simplicity, be incorporated in the hyperpolarized imaging session prior or even during the examination (Laustsen et al. 2015; Hansen et al. 2017).

The biphasic curve in Figure 2A is probably flow‐dependent; where a little amount of pyruvate enters the cells in the initial arterial phase due to the high blood flow, while a large amount of pyruvate enters the cells when the blood reaches the capillaries where low blood flow and large surface area causes a higher intracellular uptake of pyruvate.

Acute and chronic inhibition of gluconeogenesis by metformin, have been demonstrated to increase the liver and kidney lactate production. Metformin treatment was not associated with any alterations in the aerobic metabolism (bicarbonate to pyruvate ratio) in the liver (Madiraju et al. 2014; Lewis et al. 2016; Qi et al. 2018a). This is supported by previous findings in porcine kidney (second gluconeogenic tissue) following endogastric modulations with soft drinks (Laustsen et al. 2015), showing an unaltered aerobic metabolism and increased lactate production. Thus our findings support the effects of the glucose challenge in altering the metabolic demand for glucose in the liver: in turn, this change in activity leads to an altered lactate to pyruvate ratio, likely originating from a similarly altered NAD^+^ to NADH_2_ ratio.

Interestingly Le Page et al. (2016) found that T2DM mouse, induced by a high fat diet, demonstrated an increased pyruvate to tricarboxylic acid (TCA) intermediates conversion in the liver; albeit no alteration in lactate to pyruvate ratio was shown. The origin of this discrepancy is currently not known. It has been demonstrated that the main component of the ^13^C‐bicarbonate signal in healthy livers reflects the activity of PDH rather than phosphoenolpyruvate carboxykinase (PEPCK) or TCA cycle activity after pyruvate carboxylation (Jin et al. 2016) and as such represents a potential explanation for a metabolic different effects seen in the diabetic liver, where the pyruvate carboxylase is known to be altered.

The change from the fasted state to a hyperglycemic and subsequent hyperinsulinemic state causes a decrease in glycogenolysis and an increase in glycogenesis in which less lactate is converting to glucose via pyruvate (Madiraju et al. 2014; Lewis et al. 2016; Qi et al. 2018a) this is consistent with our findings of higher lactate to pyruvate ratio when gluconeogenesis decreases in which FFA also decreases while the insulin level increases. Insulin increases both lactate, alanine and bicarbonate production in the kidney of diabetic rats (Laustsen et al. 2014) as a direct insulin mediated increase in pyruvate uptake and metabolic conversion. The maintained alanine to pyruvate ratio and bicarbonate to pyruvate ratio found in this study, could be a delayed metabolic response in the porcine liver extending beyond 60 min after the glucose challenge, however this is currently unknown.

Direct translation of the association between the specific metabolic liver parameters and blood parameters could be possible in the quantification of hepatic dysfunctionality; however, similar studies of human livers are needed to establish the association. DNP has recently been translated to patients (Nelson et al. 2013; Cunningham et al. 2016). Indeed, studies on human liver metabolism based on hyperpolarization are currently ongoing but not yet published. The proposed method is not limited to glucose tolerance test and it is likely that alternative challenges test, such as hyperinsulimic clamp or oral glucose tolerances test, can also add new insights to the basic understanding of metabolic pathways, by combining the systemic hormone levels and the organ‐specific metabolic information. Thus we hypothesis that a casual dependency between typical blood parameters and the observed liver‐specific metabolic conversion following the glucose challenge exists, and secondly that this interdependency could potentially be used to improve the specificity of the hyperpolarized examination by framing the unit less conversion with clinical SI unit blood parameters.

## Limitations

It is important to note that although this study demonstrates alterations in glucose metabolism in the porcine liver, anesthesia and the consecutive injections of pyruvate volumes were necessary. Previous studies has shown a slight increase in blood parameters in control groups under prolonged anesthesia and repeated hyperpolarized measurements however not significant, a study of the pig heart with hyperpolarized MR spectroscopy showed an unaltered lactate to pyruvate ratio after 120 min of propofol anesthesia in the control group (Hansen et al. 2017) and consecutive pyruvate injections did not influence the results, as is the case in the pig kidney (Laustsen et al. 2015). However, anesthesia is likely to alter the metabolism and thus show a different response in this model than in healthy awake animals (Qi et al. 2018b). Inhalation anesthetics have been associated with alterations in glucose sensitivity due to a decrease in insulin secretion (Iwasaka et al. 1996; Tanaka et al. 2005). This may have a reduced effect on our results, which could mean that the change in lactate to pyruvate ratio in healthy animals is more pronounced. The use of 1D spectroscopy is limited to whole organ or single voxel analysis and as such future studies could use advanced imaging sequences to differentiate liver region metabolic patterns and hemodynamics responses.(Lau et al. 2010; Wiesinger et al. 2012; Schmidt et al. 2014).

The dynamic study design involves a number of unknown factors, such as changes in lactate, alanine, pyruvate and bicarbonate pools as well as changes in hormone levels. Therefore, the interpretation of our results must be done with caution, however here we show that HMRS can measure differences, especially in lactate pools, even though mechanisms behind the changes should be interpreted with causing. Measurements of the different blood parameters failed because of hyper coagulation of the blood in some of the samples; the blood was sampled in different tubes and different kits were used for measurements which may explain the difference in sample size in the different blood parameters. It was not technical possible to take blood samples while the HMRS scans were performed and therefore the blood were collected in between HMRS scans. Therefore we choose to correlate blood parameters with the subsequent HMRS data.

## Conclusion

We found that the hepatic ^13^C lactate to pyruvate ratio measured via MRS exhibits a remarkable inverse correlation with blood levels of insulin, FFA and glucagon. After a single bolus of glucose and four repeated injections of pyruvate the lactate to pyruvate ratio increased in the liver and, in the blood, levels of insulin increased while FFA and glucagon decreased. As these changes in biomarkers are directly linked to altered glucose homeostasis, our findings support hyperpolarized pyruvate as a method for in vivo quantification of glucose metabolism in the liver in a large animal model resembling human physiology. In perspective, lactate to pyruvate ratio combined with insulin, glucagon, FFA and glucose levels may be used as a diagnostic measurement to quantify the glucose utilization in the liver. Future studies should clarify to which extent pathological conditions in the liver can be evaluated by this method. This study supports the translation of hyperpolarized MRS for liver examinations by combining the GE Healthcare liver‐specific metabolism with available blood biomarkers, creating a model for the healthy liver, which could be used, noninvasively, in detection of liver‐specific metabolic pathology.

## Conflict of Interest

The authors declare no conflict of interest.

## Supporting information




**Table S1.** Physiological parameters before and after the first pyruvate injection.
**Table S2.** Raw physiological data of each pig.
**Figure S1.** Linear regression of lactate to pyruvate ratio and blood levels for individual pigs at baseline (0 min).
**Figure S2.** Linear regression of alanine to pyruvate ratio and blood levels for individual pigs at baseline (0 min).
**Figure S3.** Linear regression of bicarbonate to pyruvate ratio and blood levels for individual pigs at baseline (0 min).
**Figure S4.** The bicarbonate to pyruvate ratio and the corresponding FFA (A), glucagon (B), insulin (C) and glucose (D) at 0, 30, 50 and 70 were fitted with a single exponential regression, showing no relationship for the alanine to pyruvate ratio and FFA, glucagon, insulin and glucose respectively.
**Figure S5.** The alanine to pyruvate ratio and the corresponding FFA (A), glucagon (B), insulin (C) and glucose (D) at 0, 30, 50 and 70 were fitted with a single exponential regression, showing no relationship for the alanine to pyruvate ratio and FFA, glucagon, insulin and glucose respectively.
**Figure S6.** The lactate to pyruvate ratio and the corresponding log transformed data of FFA (A), glucagon (B), insulin (C) and glucose (D) at 0, 30, 50 and 70 was fitted with a single linear regression, showing a clear relationship for the lactate to pyruvate ratio and FFA, glucagon and insulin respectively.
