# Supplementary material for: Hyperpolarized [1‐13C] pyruvate as a possible diagnostic tool in liver disease
Source: Physiol Rep. 2018 Dec 11;6(23):e13943. doi: 10.14814/phy2.13943 (PMC6289910; doi:10.14814/phy2.13943)
Supplement: Supplementary file 1 — Table S1. Physiological parameters before and after the first pyruvate injection. Table S2. Raw physiological data of each pig. Figure S1. Linear regression of lactate to pyruvate ratio and blood levels for individual pigs at baseline (0 min). Figure S2. Linear regression of alanine to pyruvate ratio and blood levels for individual pigs at baseline (0 min). Figure S3. Linear regression of bicarbonate to pyruvate ratio and blood levels for individual pigs at baseline (0 min). Figure S4. The bicarbonate to pyruvate ratio and the corresponding FFA (A), glucagon (B), insulin (C) and glucose (D) at 0, 30, 50 and 70 were fitted with a single exponential regression, showing no relationship for the alanine to pyruvate ratio and FFA, glucagon, insulin and glucose respectively. Figure S5. The alanine to pyruvate ratio and the corresponding FFA (A), glucagon (B), insulin (C) and glucose (D) at 0, 30, 50 and 70 were fitted with a single exponential regression, showing no relationship for the alanine to pyruvate ratio and FFA, glucagon, insulin and glucose respectively. Figure S6. The lactate to pyruvate ratio and the corresponding log transformed data of FFA (A), glucagon (B), insulin (C) and glucose (D) at 0, 30, 50 and 70 was fitted with a single linear regression, showing a clear relationship for the lactate to pyruvate ratio and FFA, glucagon and insulin respectively. [file PHY2-6-e13943-s001.docx]

**Supplementary**

**Hyperpolarized [1-^13^C] pyruvate as a possible diagnostic tool in liver disease**

Uffe Kjærgaard^1^, Christoffer Laustsen^1^, Thomas Nørlinger^1^, Rasmus Stilling Tougaard^1,2^, Emmeli Mikkelsen^1^, Haiyun Qi^1^, Lotte Bonde Bertelsen^1^, Niels Jessen^3,4^ and Hans Stødkilde-Jørgensen^1^

^1^*MR Research Centre, Aarhus University Hospital, Aarhus, DK*

*^2^Department of Cardiology, Aarhus University Hospital, Aarhus, DK*

*^3^Department of Biomedicine, Aarhus University*

*^4^Department of Clinical Pharmacology, Aarhus University Hospital*

**Table 1. Physiological parameters before and after the first pyruvate injection**

|  | **0 min ± SEM** | **10 min ± SEM** | **Subjects** |
| --- | --- | --- | --- |
| Weight (kg) | 30.9 ± 0.38 | - | N = 11 |
| Heart rate (bpm) | 67 ± 4.1 | 71 ± 3.0 | N = 11 |
| Systolic BP (mm/Hg) | 101 ± 4.7 | 109 ± 5.1 | N = 11 |
| Diastolic BP (mm/Hg) | 51 ± 3.4 | 53 ± 3.6 | N = 11 |

**Table 1. Legend:** Baseline values for pigs before and after the first hyperpolarization measurement. Values are given as mean ± SEM. Blood pressure (BP).

**Table 2.** Raw physiological data of each pig.

| **Subject #** | **1** |  | **Bodyweight [kg]** | **31.9** |  |
| --- | --- | --- | --- | --- | --- |
|  |  |  |  |  |  |
| Time [min] | Lactate [mmol/l] | Glucose [mmol/l] | Insulin [pmol/l] | Glucagon [pmol/l] | FFA [mmol/l] |
| 0 | NA | 3.2 | NA | NA | 0.033 |
| 4 | NA | 3.8 | NA | NA | 0.049 |
| 10 | NA | 29 | NA | NA | 0.042 |
| 30 | NA | 17.1 | 11 | 1.57 | 0.024 |
| 50 | NA | 13.8 | 19 | 1.542 | 0.024 |
| 70 | NA | 11.4 | 46 | 1.346 | 0.013 |
|  |  |  |  |  |  |
| **Subject #** | **2** |  | **Bodyweight [kg]** | **32.35** |  |
|  |  |  |  |  |  |
| Time [min] | Lactate [mmol/l] | Glucose [mmol/l] | Insulin [pmol/l] | Glucagon [pmol/l] | FFA [mmol/l] |
| 0 | 0.6 | 3.5 | 4 | 2.361 | 0.046 |
| 4 | 0.9 | 3.5 | NA | NA | 0.039 |
| 10 | 0.7 | 27 | NA | NA | NA |
| 30 | 1 | 15.6 | 123 | 1.57 | NA |
| 50 | 1.1 | 12.6 | 171 | 1.682 | NA |
| 70 | 1 | 9.7 | 239 | NA | NA |
|  |  |  |  |  |  |
| **Subject #** | **3** |  | **Bodyweight [kg]** | **31.25** |  |
|  |  |  |  |  |  |
| Time [min] | Lactate [mmol/l] | Glucose [mmol/l] | Insulin [pmol/l] | Glucagon [pmol/l] | FFA [mmol/l] |
| 0 | 0.7 | 5.2 | NA | NA | 0.051 |
| 4 | 1 | 5.1 | NA | NA | 0.065 |
| 10 | 0.8 | 29 | NA | NA | 0.053 |
| 30 | 1.2 | 17.1 | 26 | 1.88 | 0.042 |
| 50 | 1.3 | 13.9 | 44 | NA | 0.035 |
| 70 | 1.2 | 11.7 | 90 | NA | 0.023 |
|  |  |  |  |  |  |
| **Subject #** | **4** |  | **Bodyweight [kg]** | **29.05** |  |
|  |  |  |  |  |  |
| Time [min] | Lactate [mmol/l] | Glucose [mmol/l] | Insulin [pmol/l] | Glucagon [pmol/l] | FFA [mmol/l] |
| 0 | 1.3 | 5 | 45 | 5.072 | 0.064 |
| 4 | 1.6 | 5 | 59 | NA | 0.064 |
| 10 | 1.4 | 30 | 109 | NA | 0.044 |
| 30 | NA | 18.1 | 148 | 2.875 | 0.042 |
| 50 | 1.9 | 14 | 172 | 2.817 | 0.031 |
| 70 | 1.5 | 11.7 | 287 | NA | 0.031 |
|  |  |  |  |  |  |
| **Subject #** | **5** |  | **Bodyweight [kg]** | **30.6** |  |
|  |  |  |  |  |  |
| Time [min] | Lactate [mmol/l] | Glucose [mmol/l] | Insulin [pmol/l] | Glucagon [pmol/l] | FFA [mmol/l] |
| 0 | 1.1 | 4.9 | 5 | 1.514 | 0.129 |
| 4 | 1.4 | 5.2 | 9 | NA | 0.079 |
| 10 | 1.3 | 30 | 32 | NA | 0.065 |
| 30 | 1.4 | 17.4 | 75 | 1.151 | 0.053 |
| 50 | 1.6 | 14.2 | 97 | 1.123 | 0.042 |
| 70 | 1.7 | 11.9 | 112 | 1.123 | 0.028 |
|  |  |  |  |  |  |
| **Subject #** | **6** |  | **Bodyweight [kg]** | **32.45** |  |
|  |  |  |  |  |  |
| Time [min] | Lactate [mmol/l] | Glucose [mmol/l] | Insulin [pmol/l] | Glucagon [pmol/l] | FFA [mmol/l] |
| 0 | 1.2 | 4.9 | 8 | NA | 0.134 |
| 4 | 1.6 | 6.8 | 7 | NA | 0.139 |
| 10 | 1.4 | 29 | 16 | NA | 0.095 |
| 30 | 1.7 | 21.4 | 34 | NA | 0.051 |
| 50 | 1.9 | 16.5 | 67 | NA | 0.035 |
| 70 | 1.9 | 13.7 | 112 | NA | 0.025 |
|  |  |  |  |  |  |
| **Subject #** | **7** |  | **Bodyweight [kg]** | **30.5** |  |
|  |  |  |  |  |  |
| Time [min] | Lactate [mmol/l] | Glucose [mmol/l] | Insulin [pmol/l] | Glucagon [pmol/l] | FFA [mmol/l] |
| 0 | 1 | 3.2 | 12 | NA | 0.135 |
| 4 | 1.2 | 2.9 | 14 | NA | 0.115 |
| 10 | 1.1 | 26 | 70 | NA | 0.103 |
| 30 | 1.1 | 15.9 | 105 | NA | 0.108 |
| 50 | 1.3 | 13.4 | 244 | NA | 0.083 |
| 70 | 1.5 | 11.9 | 466 | NA | 0.029 |
|  |  |  |  |  |  |
| **Subject #** | **8** |  | **Bodyweight [kg]** | **30.8** |  |
|  |  |  |  |  |  |
| Time [min] | Lactate [mmol/l] | Glucose [mmol/l] | Insulin [pmol/l] | Glucagon [pmol/l] | FFA [mmol/l] |
| 0 | 1.2 | NA | 17 | 2.617 | 0.324 |
| 4 | 1.6 | 7.1 | 15 | NA | 0.348 |
| 10 | 1.4 | 33 | NA | NA | 0.267 |
| 30 | 1.7 | 20.6 | NA | 2.446 | 0.188 |
| 50 | NA | 17.3 | NA | NA | 0.012 |
| 70 | 1.8 | 18 | NA | 2.39 | 0.026 |
|  |  |  |  |  |  |
| **Subject #** | **9** |  | **Bodyweight [kg]** | **32** |  |
|  |  |  |  |  |  |
| Time [min] | Lactate [mmol/l] | Glucose [mmol/l] | Insulin [pmol/l] | Glucagon [pmol/l] | FFA [mmol/l] |
| 0 | 1 | 4.1 | 7 | 2.106 | 0.326 |
| 4 | 1.3 | 4.2 | 6 | NA | 0.346 |
| 10 | 1.1 | 29 | 33 | NA | 0.244 |
| 30 | 1.8 | 17.6 | 112 | NA | 0.182 |
| 50 | 2.2 | 14.3 | 138 | NA | 0.18 |
| 70 | 2.1 | 11.5 | 161 | 1.654 | 0.095 |
|  |  |  |  |  |  |
| **Subject #** | **10** |  | **Bodyweight [kg]** | **29.6** |  |
|  |  |  |  |  |  |
| Time [min] | Lactate [mmol/l] | Glucose [mmol/l] | Insulin [pmol/l] | Glucagon [pmol/l] | FFA [mmol/l] |
| 0 | 1.2 | 5.4 | 10 | NA | 0.33 |
| 4 | 1.4 | 5.3 | 6 | NA | 0.25 |
| 10 | 1.3 | 28 | 36 | NA | 0.157 |
| 30 | 1.8 | 18.1 | 89 | NA | 0.18 |
| 50 | 2.1 | 15.5 | 116 | NA | 0.162 |
| 70 | 2 | 12.4 | 256 | NA | 0.122 |
|  |  |  |  |  |  |
| **Subject #** | **11** |  | **Bodyweight [kg]** | **28.95** |  |
|  |  |  |  |  |  |
| Time [min] | Lactate [mmol/l] | Glucose [mmol/l] | Insulin [pmol/l] | Glucagon [pmol/l] | FFA [mmol/l] |
| 0 | 1 | 5.4 | 30 | NA | 0.473 |
| 4 | 1.3 | 5.6 | 49 | NA | 0.471 |
| 10 | 1.3 | 21.9 | 367 | NA | 0.238 |
| 30 | 1.8 | 16.1 | 329 | NA | 0.291 |
| 50 | 2.2 | 12.9 | 292 | NA | 0.127 |
| 70 | 2.3 | 10.4 | 373 | NA | 0.203 |
|  |  |  |  |  |  |

**Table 2.** Raw hyperpolarized MRI data of each pig.

| **Subject #** | **1** |  |  |
| --- | --- | --- | --- |
|  |  |  |  |
| Time [min] | Lactate/pyruvate | Alanine/pyruvate | Bicarbonate/pyruvate |
| 0 | 12.49 | 7.75 | 0.18 |
| 20 | 8.85 | 3.91 | 0.2 |
| 40 | 22.54 | 6.27 | 0.43 |
| 60 | 25.01 | 4.02 | 0.85 |
|  |  |  |  |
| **Subject #** | **2** |  |  |
|  |  |  |  |
| Time [min] | Lactate/pyruvate | Alanine/pyruvate | Bicarbonate/pyruvate |
| 0 | 6.55 | 3.97 | 0.09 |
| 20 | 9.05 | 3.51 | 0.3 |
| 40 | 10.54 | 2.62 | 0.38 |
| 60 | 25.39 | 4.1 | 0.51 |
|  |  |  |  |
| **Subject #** | **3** |  |  |
|  |  |  |  |
| Time [min] | Lactate/pyruvate | Alanine/pyruvate | Bicarbonate/pyruvate |
| 0 | 12.94 | 5.09 | 0.21 |
| 20 | 13.85 | 3.97 | 0.21 |
| 40 | 22.36 | 2.61 | 0.43 |
| 60 | 18.73 | 5.02 | 0.66 |
|  |  |  |  |
| **Subject #** | **4** |  |  |
|  |  |  |  |
| Time [min] | Lactate/pyruvate | Alanine/pyruvate | Bicarbonate/pyruvate |
| 0 | 15,29 | 3.13 | 0.3 |
| 20 | 12,19 | 3.95 | 0.4 |
| 40 | 11,09 | 2.85 | 0.13 |
| 60 | NA | NA | NA |
|  |  |  |  |
| **Subject #** | **5** |  |  |
|  |  |  |  |
| Time [min] | Lactate/pyruvate | Alanine/pyruvate | Bicarbonate/pyruvate |
| 0 | 9.86 | 1.59 | 0.23 |
| 20 | 8.74 | 2.6 | 0.12 |
| 40 | 11.06 | 5.62 | 0.16 |
| 60 | 10.27 | 3.18 | 0.14 |
|  |  |  |  |
| **Subject #** | **6** |  |  |
|  |  |  |  |
| Time [min] | Lactate/pyruvate | Alanine/pyruvate | Bicarbonate/pyruvate |
| 0 | 5.1 | 1.2 | 0.15 |
| 20 | 6.08 | 1.49 | 0.18 |
| 40 | 6.04 | 1.47 | 0.14 |
| 60 | 7.2 | 2.1 | 0.19 |
|  |  |  |  |
| **Subject #** | **7** |  |  |
|  |  |  |  |
| Time [min] | Lactate/pyruvate | Alanine/pyruvate | Bicarbonate/pyruvate |
| 0 | 5.26 | 4.15 | 0.32 |
| 20 | NA | NA | NA |
| 40 | 6.69 | 1.42 | 1.7 |
| 60 | 8.83 | 1.15 | 0.19 |
|  |  |  |  |
| **Subject #** | **8** |  |  |
|  |  |  |  |
| Time [min] | Lactate/pyruvate | Alanine/pyruvate | Bicarbonate/pyruvate |
| 0 | 12.12 | 2.88 | 0.33 |
| 20 | 12.41 | 2.06 | 0.27 |
| 40 | 11.58 | 1.88 | 0.43 |
| 60 | 14.85 | 3.88 | 0.49 |
|  |  |  |  |
| **Subject #** | **9** |  |  |
|  |  |  |  |
| Time [min] | Lactate/pyruvate | Alanine/pyruvate | Bicarbonate/pyruvate |
| 0 | 11.46 | 2.64 | 0.55 |
| 20 | 17.71 | 3.57 | 0.4 |
| 40 | 17.41 | 3.06 | 0.25 |
| 60 | 24.48 | 4.9 | 0.31 |
|  |  |  |  |
| **Subject #** | **10** |  |  |
|  |  |  |  |
| Time [min] | Lactate/pyruvate | Alanine/pyruvate | Bicarbonate/pyruvate |
| 0 | 13.03 | 1.99 | 0.36 |
| 20 | 12.2 | 4.43 | 0.1 |
| 40 | 13.07 | 1.41 | 0.06 |
| 60 | 12.39 | 1.75 | 0.14 |
|  |  |  |  |
| **Subject #** | **11** |  |  |
|  |  |  |  |
| Time [min] | Lactate/pyruvate | Alanine/pyruvate | Bicarbonate/pyruvate |
| 0 | 6.2 | 2.61 | 0.1 |
| 20 | 10.2 | 3.06 | 1.08 |
| 40 | 7.8 | 1.39 | 0.51 |
| 60 | 10.38 | 5.07 | 0.05 |

**
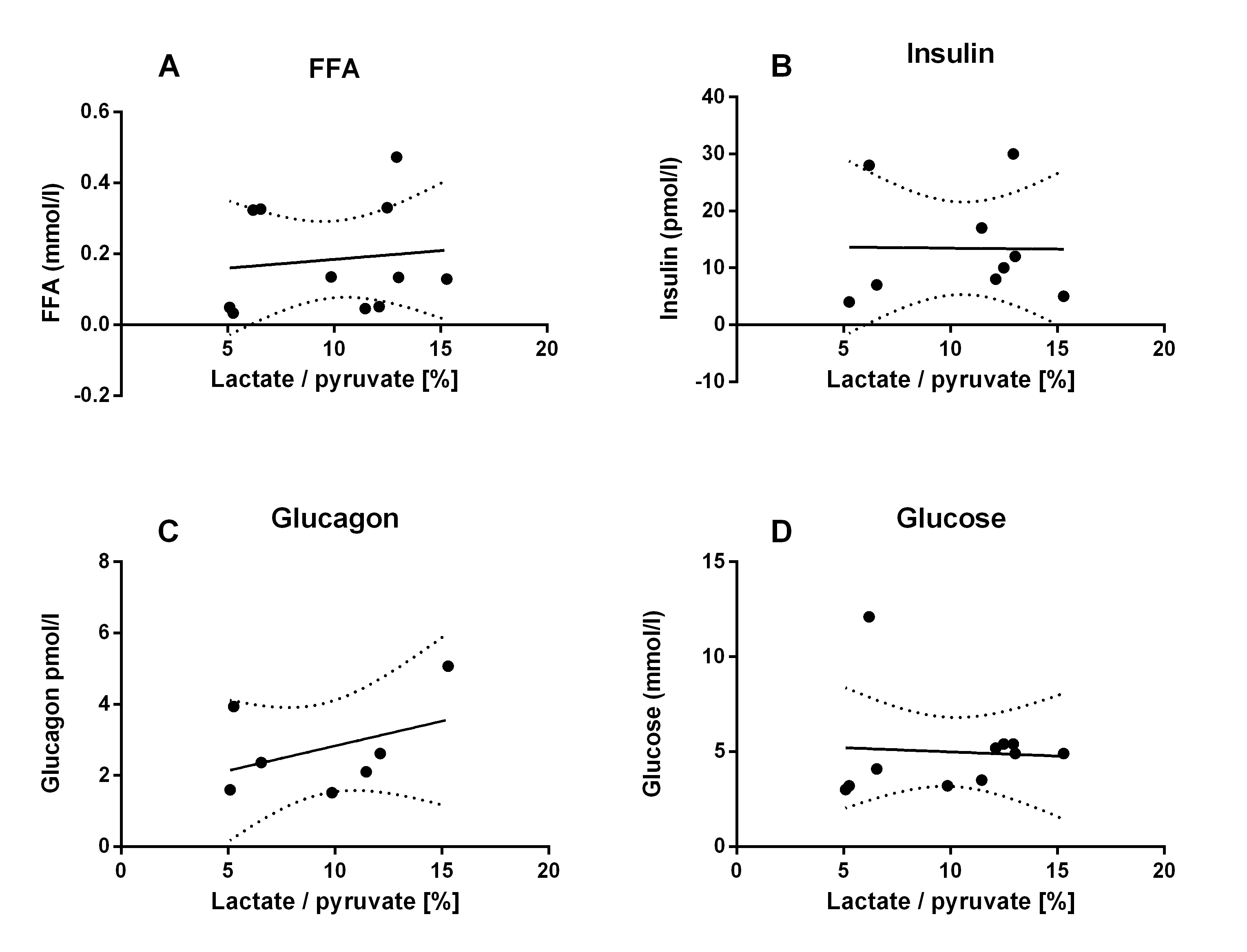
Supplementary Fig. 1:** Linear regression of lactate to pyruvate ratio and blood levels for individual pigs at baseline (0 min). FFA (A), insulin (B), glucagon (C) and glucose (D). No significant linear regressions were found.

**
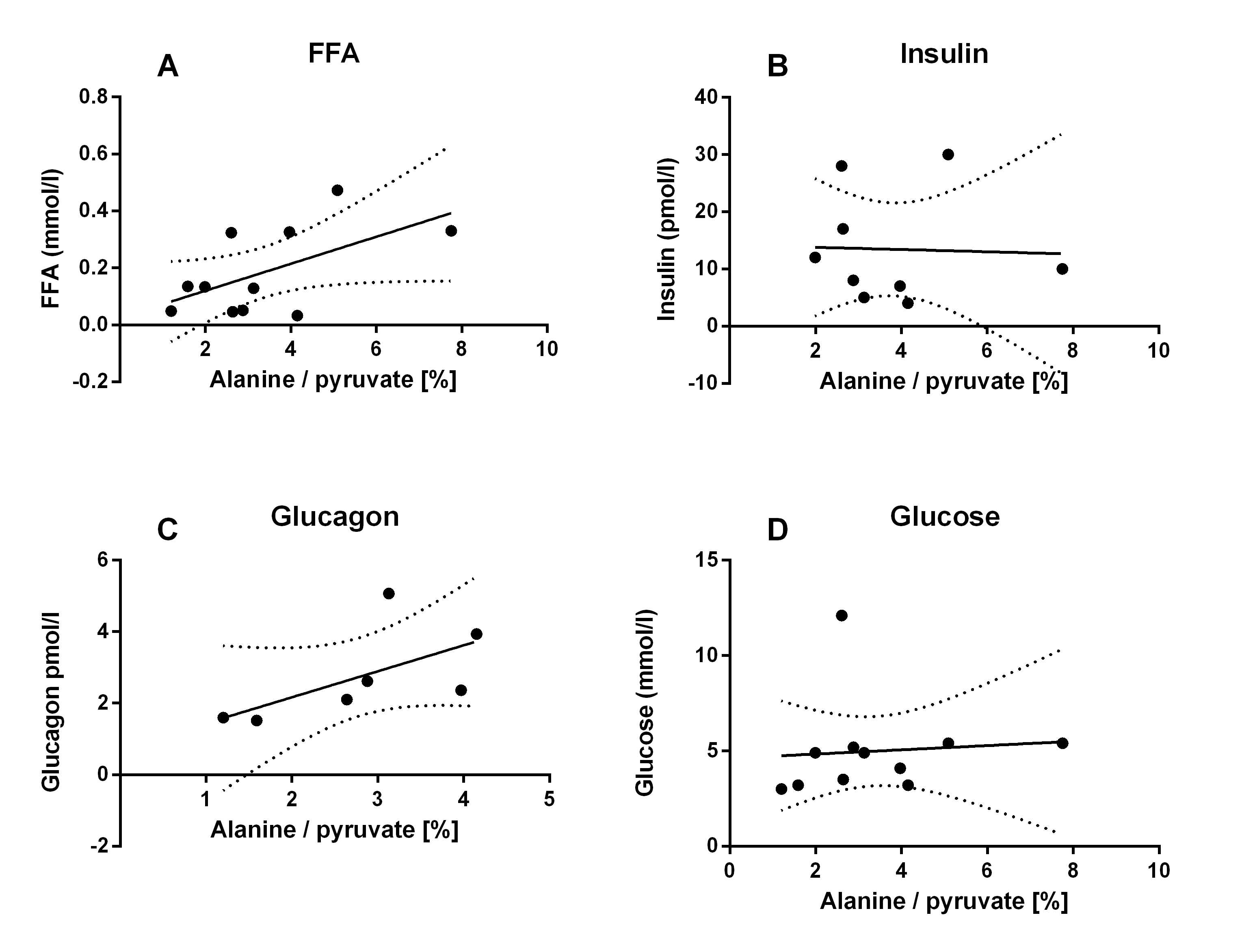
**

**Supplementary Fig. 2:** Linear regression of alanine to pyruvate ratio and blood levels for individual pigs at baseline (0 min). FFA (A), insulin (B), glucagon (C) and glucose (D). No significant linear regressions were found.

**
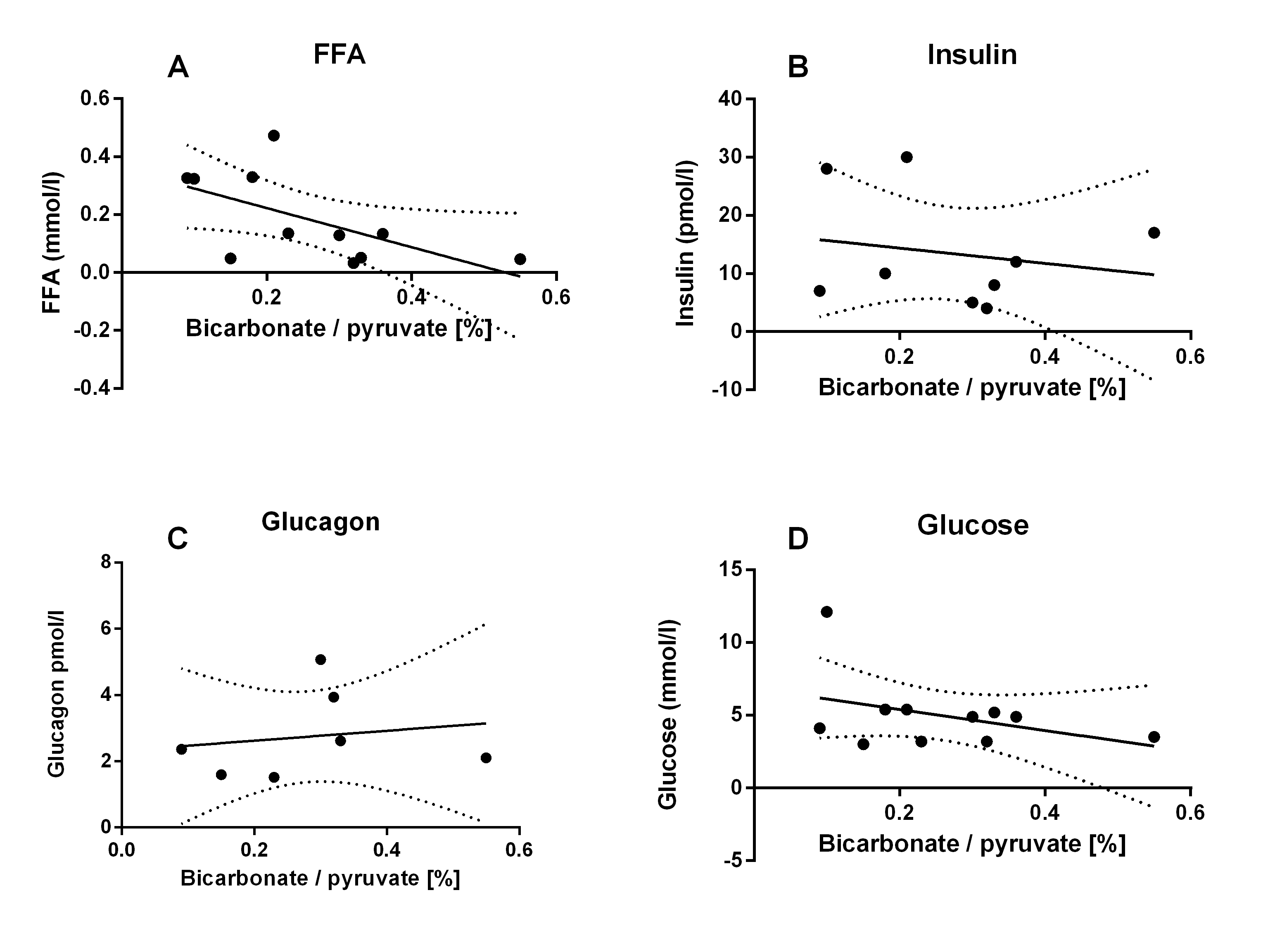
**

**Supplementary Fig. 3:** Linear regression of bicarbonate to pyruvate ratio and blood levels for individual pigs at baseline (0 min). FFA (A), insulin (B), glucagon (C) and glucose (D). No significant linear regressions were found.

**
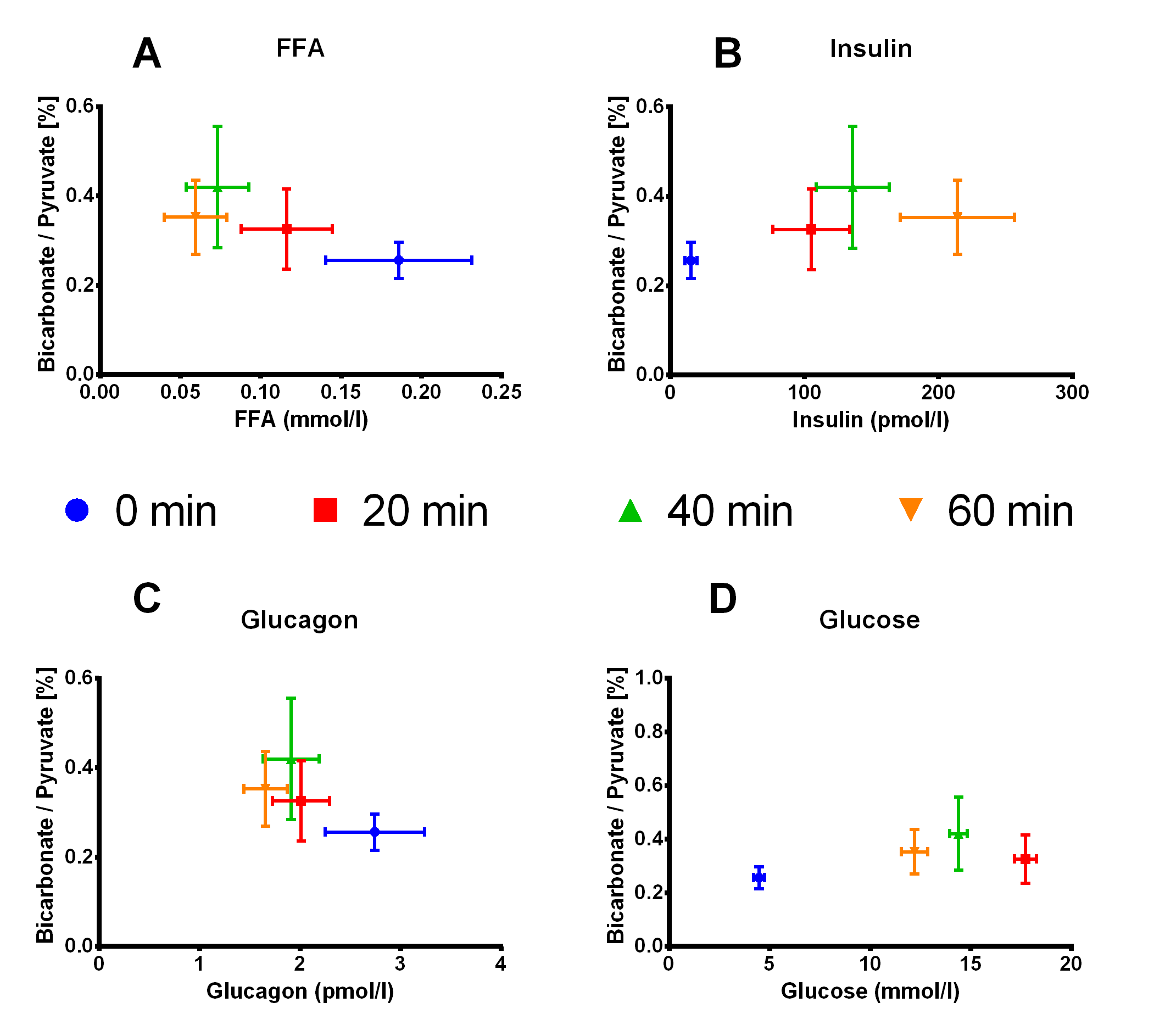
**

**Supplementary Figure 4:** The bicarbonate to pyruvate ratio and the corresponding FFA (A), glucagon (B), insulin (C) and glucose (D) at 0, 30, 50 and 70 were fitted with a single exponential regression, showing no relationship for the alanine to pyruvate ratio and FFA, glucagon, insulin and glucose respectively.

**
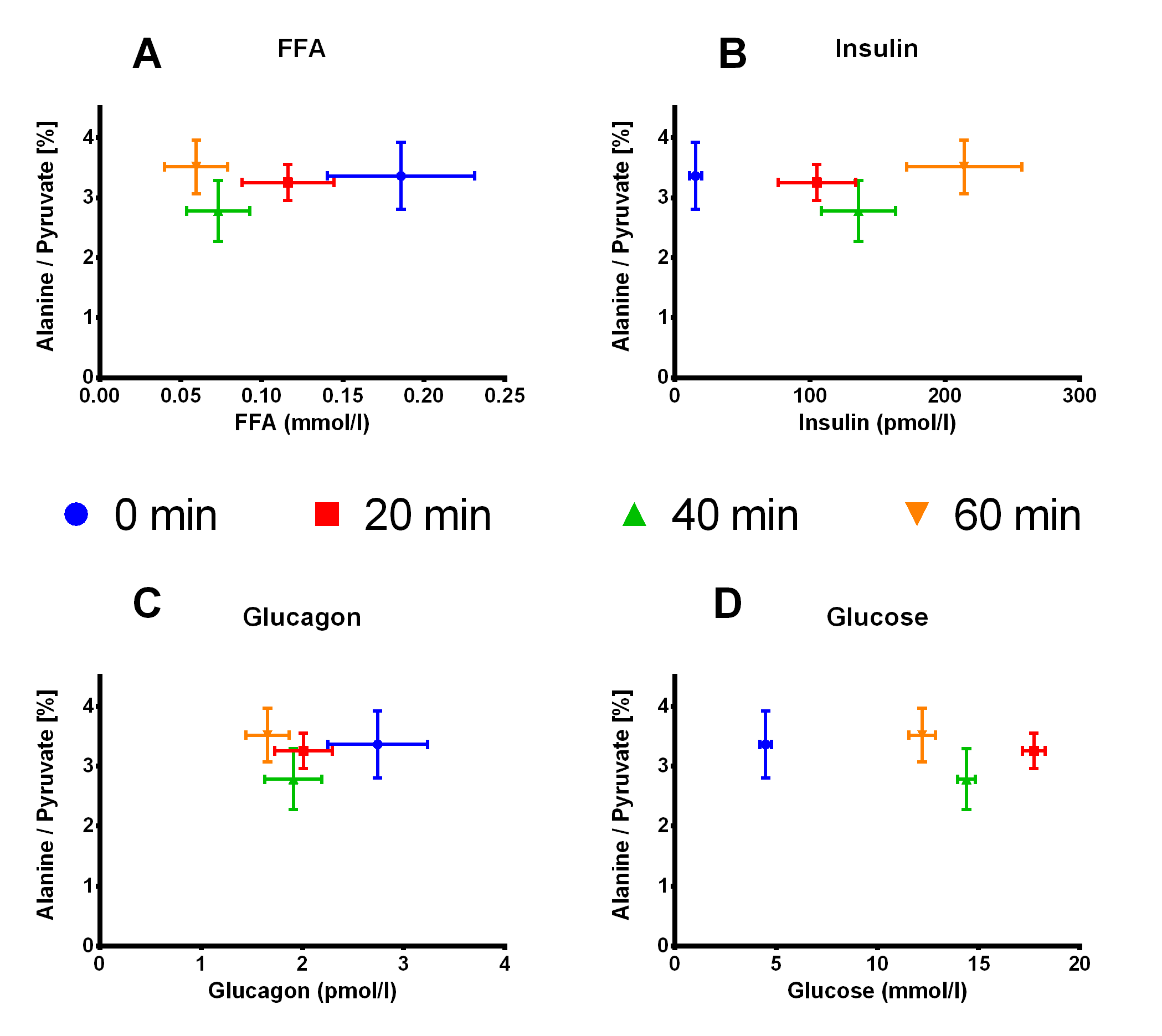
**

**Supplementary Fig. 5:** The alanine to pyruvate ratio and the corresponding FFA (A), glucagon (B), insulin (C) and glucose (D) at 0, 30, 50 and 70 were fitted with a single exponential regression, showing no relationship for the alanine to pyruvate ratio and FFA, glucagon, insulin and glucose respectively.





**Supplementary Fig 6.** The lactate to pyruvate ratio and the corresponding log transformed data of FFA (A), glucagon (B), insulin (C) and glucose (D) at 0, 30, 50 and 70 was fitted with a single linear regression, showing a clear relationship for the lactate to pyruvate ratio and FFA, glucagon and insulin respectively. A similar dependency was observed by omitting the initial mean for glucose. The color code represents the time of injection of hyper polarized ^13^C pyruvate injection: blue (0 min.), red (20 min.), green (40 min.), and orange (60 min.). Sample size for glucose (n= 11), insulin (n=9), glucagon (n=7) FFA (n=10).





**Supplementary Fig 6.**

The lactate to pyruvate ratio and the corresponding log transformed data of blood lactate at 0, 30, 50 and 70 min (n=11) was fitted with a single linear regression, showing a clear relationship for the lactate to pyruvate ratio to blood lactate (p=0.0495).
